# Multifunctional Three-in-One Sensor on t-ZnO for Ultraviolet and VOC Sensing for Bioengineering Applications

**DOI:** 10.3390/bios14060293

**Published:** 2024-06-05

**Authors:** Rajat Nagpal, Cristian Lupan, Adrian Bîrnaz, Alexandr Sereacov, Erik Greve, Monja Gronenberg, Leonard Siebert, Rainer Adelung, Oleg Lupan

**Affiliations:** 1 Center for Nanotechnology and Nanosensors, Department of Microelectronics and Biomedical Engineering, Technical University of Moldova, 168 Stefan cel Mare Av., MD-2004 Chisinau, Moldova; cristian.lupan@mib.utm.md (C.L.); adrian.birnaz@mib.utm.md (A.B.); alexandr.sereacov@mib.utm.md (A.S.); 2 Department of Materials Science, Functional Nanomaterials, Faculty of Engineering, Kiel University, Kaiserstraße 2, D-24143 Kiel, Germany; ergr@tf.uni-kiel.de (E.G.); momo@tf.uni-kiel.de (M.G.); ra@tf.uni-kiel.de (R.A.)

**Keywords:** ZnO tetrapodal networks, selectivity, rejection ratio, optoelectronics, gas sensor

## Abstract

Zinc oxide (ZnO) is considered to be one of the most explored and reliable sensing materials for UV detection due to its excellent properties, like a wide band gap and high exciton energy. Our current study on a photodetector based on tetrapodal ZnO (t-ZnO) reported an extremely high UV response of ~9200 for 394 nm UV illumination at 25 °C. The t-ZnO network structure and morphology were investigated using XRD and SEM. The sensor showed a UV/visible ratio of ~12 at 25 °C for 394 nm UV illumination and 443 nm visible illumination. By increasing the temperature, monotonic decreases in response and recovery time were observed. By increasing the bias voltage, the response time was found to decrease while the recovery time was increased. The maximum responsivity shifted to higher wavelengths from 394 nm to 400 nm by increasing the operating temperature from 25 °C to 100 °C. The t-ZnO networks exhibited gas-sensing performances at temperatures above 250 °C, and a maximum response of ~1.35 was recorded at 350 °C with a good repeatability and fast recovery in 16 s for 100 ppm of *n*-butanol vapor. This study demonstrated that t-ZnO networks are good biosensors that can be used for diverse biomedical applications like the sensing of VOCs (volatile organic compounds) and ultraviolet detection under a wide range of temperatures, and may find new possibilities in biosensing applications.

## 1. Introduction

ZnO is a direct bandgap and n-type semiconductor, making it a suitable candidate for photodetection applications [1]. Ultraviolet (UV) optoelectronics is one of the most explored research fields for ZnO. UV detectors are extensively used in diverse applications such as aerospace [2], military applications [3], biomedical [4], environmental monitoring [5], and space applications [6], etc. Ultraviolet radiation has both positive (vitamin D production) and negative impacts on human health (skin aging, skin burning, or skin cancer) [7,8]. Skin cancer types like basal cell carcinoma [9] or melanoma [10] may be caused by overexposure to UV-A or UV-B irradiations. They may mutate the gene sequence or harm DNA, which may lead to skin cancer or other skin disorders [11]. Melanin enzyme production is controlled by the Melanocortin 1 receptor (MC1R). Weak signaling may result in an insufficient production of melanin, which depends on various genetic factors such as skin color and hair color alleles [11]. Thus, the continuous monitoring of UV exposure may collect personalized real-time data and good sensors can detect associated signals that can help to maintain regular UV exposure for well-being.

Many studies have focused on the detection of weak UV signals with low optical power in the order of a few mW [12,13]. Weak UV signal detection can help with the development of diagnostic tools for certain diseases like cancer by monitoring changes in the fluorescence properties of biological tissues [14]. In drug development process, weak UV signal detection is used for screening and evaluating the efficacy of drugs in molecular assays [15]. There have been several studies on multifunctional sensors to detect different signals on a single chip [16,17]. Chakraborty et al. [18] demonstrated a two-in-one sensor based on metal–organic-framework-coated ZnO for UV and hydrogen sensing. Xu et al. [19] demonstrated a triboelectric sensor used as a tactile sensor and motion monitor for robotic applications. ZnO is a highly explored functional material used for various applications, including UV sensing [20], VOC and gas sensing [21], photocatalysis [22], piezoelectricity [23], solar cells [24], and triboelectricity [25]. t-ZnO (tetrapodal zinc oxide) has remarkably long exciton photoluminescence lifetimes, which can be attributed to the single-crystallinity and low defect density of t-ZnO. These properties make t-ZnO remarkable among all other versions of ZnO [26]. Adelung et al. demonstrated [27] the rapid fabrication of interconnected t-ZnO networks using B-FTS (Burner Flame Transport Synthesis), which is the most efficient synthesis approach to directly pattern substrates. Furthermore, the applications of tetrapodal networks have been extended to temperature sensing, VOC detection, and photodetection at different temperatures (25 °C to 100 °C) and different UV-A wavelengths (394 nm, 400 nm, and 443 nm) for biomedical purposes.

There have been many advances in ZnO-based photodetection due to its excellent signal-to-noise ratio, low dark current, high quantum efficiency, and high UV response under various operating conditions [28,29]. The requirements for a high signal-to-noise ratio are a high voltage supply and filters to eliminate low-energy interfering signals. Due to the wide band gap energy of ZnO, it has a high thermal stability, allowing for its operation in adverse environments. Many studies have reported [30,31] that, as the operating temperature increases, the signal-to-noise ratio decreases due to various reasons such as increases in dark current or thermal noise, etc. Sharma et al. reported [32] the impact of temperature on the signal-to-noise ratio of optical-fiber-based sensors. The authors explained the thermal effect on the signal-to-noise ratio within the temperature range from 300 to 600 K. It was shown that there is a monotonic decrease in the signal-to-noise ratio for this temperature range, for both the positive and negative thermo-optic coefficients of the sensing material. These reported studies demonstrate the direct effect of temperature on the signal-to-noise ratio, affecting the sensitivity, stability, and other sensing properties. At elevated temperatures, an unwanted leakage current arises for semiconductors, which increases the noise.

The detection of n-butanol is important for assessing its effects on organisms and for the monitoring of fermentation processes. In the field of bioengineering, protein folding equilibrium and the effect of different alcohol groups on protein hydrophobicity have been studied [33]. Alcohol exposure can impact cell viability and proliferation. Therefore, the detection of different alcohol groups is required to assess the effects of different alcohol concentrations on the overall health and growth of cultured cells. High doses of n-butanol can lead to apoptosis [34]. There are many studies on the alterations in hydrophobicity and molecular integrity in the presence of n-propanol and other alcohols [35,36]. The extent of alteration due to different alcohols is emphasized in these studies. Other applications like biological fluids need to be thermally monitored for lab-on-chip applications. During lab-on-chip microfluidic biological experiments, the fast monitoring of temperature is required to preserve biological samples [37]. In dermatology, UV cameras are needed to diagnose and monitor skin disorders. Wearable sensors based on ZnO can monitor and record daily exposure to UV rays, which can then be used to regulate exposure during routine activities [38]. Long-term UV-A monitoring and regulation can prevent wrinkles and possibly skin cancer [39]. There is high demand for a biosensor that is selective in all types of bio-applications [40].

**Figure 1:**
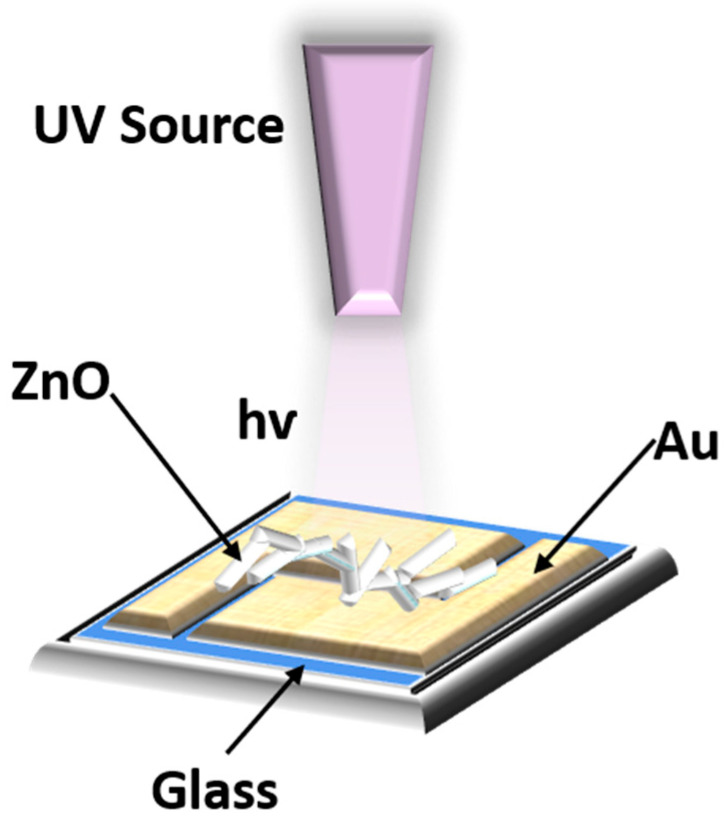
Schematic diagram of UV illumination on Au contacted t-ZnO networks.

Our current study focuses on multifunctional sensors based on t-ZnO networks for UV-A (394 nm and 400 nm) detection, VOC sensing, and temperature sensing. This study reports t-ZnO networks mounted between Au-contacts (Figure 1) as good temperature sensors with a sensitivity of 0.42%/°C. Furthermore, selective VOC sensing at elevated temperatures (300 °C and 350 °C) and continuous efficient UV-A monitoring at temperatures between 25 °C and 100 °C is demonstrated. The fundamental information about oxygen interstitials is also extracted by calculating the corresponding activation energy (~0.4 eV) from the Arrhenius plot. Due to the tetrapod structure of the ZnO, the novelty of this work is that the fabricated ZnO network-based photodetector exhibits a high UV responsivity (~9200) at low-power photo illumination (~4.1 µW) at 25 °C, which can be used in wearable sensors for the continuous monitoring of UV exposure for medical data recording and many other applications.

## 2. Experimental

### 2.1. Materials and Methods

#### 2.1.1. Preparation of t-ZnO Networks and Sensor Setup

t-ZnO networks were obtained using flame transport synthesis (FTS) developed by Mishra et al. by using PVB powder as a sacrificial polymer, as reported before in [41,42]. In a simple combustion process, Zn microparticles and PVB (1:2), respectively, formed a ZnO tetrapod in the presence of oxygen. The mixture of Zn and PVB powder was heated to 900 °C for 30 min, resulting in the formation of ZnO microparticles. During this process, the sacrificial polymer (PVB) burnt instead of the Zn, allowing it to reach elevated temperatures without combustion to attain the t-ZnO networks. As the PVB burnt away completely, Zn vaporized due to the high vapor pressure of Zn, resulting in the formation of ZnO. For sensing applications, ZnO tetrapods were placed on a glass substrate with gold contacts, as shown in Figure 1. The t-ZnO and the Au contacts were electrically connected by using silver paste (Figure 1).

#### 2.1.2. Material Characterization

**Figure 2:**
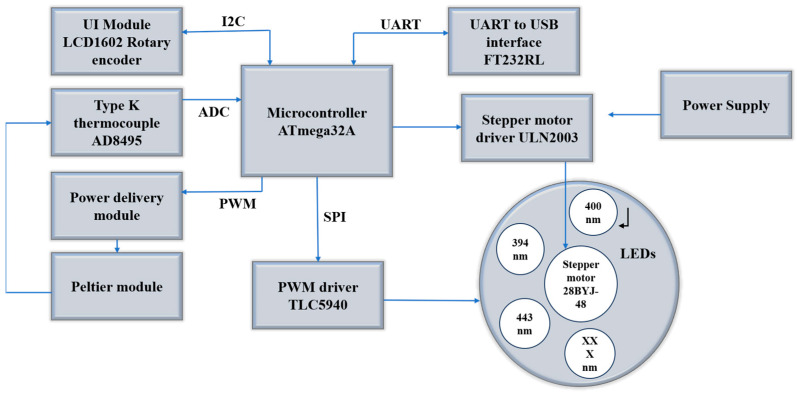
The schematic diagram for UV testing setup.

The morphological, structural, and gas-sensing characteristics of the ZnO tetrapod networks were investigated using scanning electron microscopy (SEM, Carl Zeiss, Oberkochen, Germany) and X-ray diffraction, as previously reported in our work [43]. UV measurements were performed using a custom-built setup with a Keithley 2450 multimeter (Keithley, Cleveland, OH, USA) and heating chamber in normal ambient conditions, as shown in Figure 2.

#### 2.1.3. UV Installation

The measurement setup for UV detection consisted of different components such as LEDs in the UV-A wavelength range and the visible range, which were placed on an aluminum-printed circuit board (PCB). A rotary motion of the board was controlled by a stepper motor, which controlled the position of the sample under test just below the selected LED, as shown in the bottom right of the test setup diagram in Figure 2. The default position was marked by the arrow on the rotary when no LED was manually selected. The intensity of the light illumination was well controlled by the TLC5940 PWM (Texas Instruments, Dallas, TX, USA) (pulse width modulation) driver. The PWM operating frequency was about 2 kHz. A Peltier element was used to provide an operating temperature range from −10 to 100 °C, controlled by a power MOSFET module with a feedback mechanism. This feedback signal was captured through a K-type thermocouple amplified by AD8495 (Adafruit industries, New York, NY, USA) and fed back to the microcontroller through an ADC input.

All LEDs (394 nm, 400 nm, and 443 nm wavelengths, respectively) were calibrated, and their optical power was measured at different power settings using a Newport power meter (model:843-R) (Newport Corporation, Irvine, CA, USA). Using a graph analysis, all sources were calibrated to the same optical power, with their respective photo-illuminations controlled within the illumination range (min. to max. in %).

The whole measurement was carried out in constant voltage mode and the data were acquired by a Keithley 2450. The entire apparatus, shown schematically in Figure 2, is well established and was fully controlled by specialized software [44].

## 3. Results and Discussion

### 3.1. Morphological and Structural Characterization

**Figure 3:**
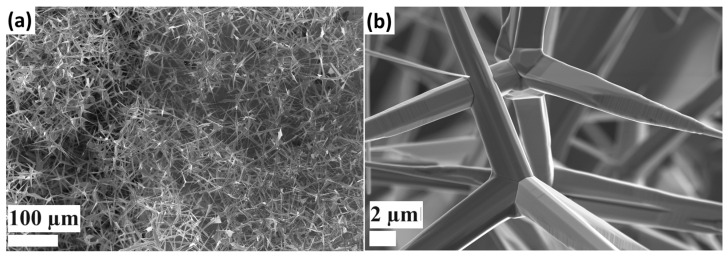
SEM images of t-ZnO networks were obtained using the FTS method at different magnifications: (**a**) lower magnification, (**b**) interconnected networks at higher magnification.

SEM images of the t-ZnO networks grown using the FTS method are presented in Figure 3, where an interconnected network of numerous ZnO tetrapods is observed. Interconnected tetrapods have advantages like self-assembly for efficient sensing applications [45]. At a higher magnification (Figure 3b), it was observed that the t-ZnO had a variable diameter of ~2 µm at the base. The central crystalline core enhances the 3D-shape retention of the tetrapodal arm under harsh conditions and enables reliable electrical contacts for sensing applications [45,46].

**Figure 4:**
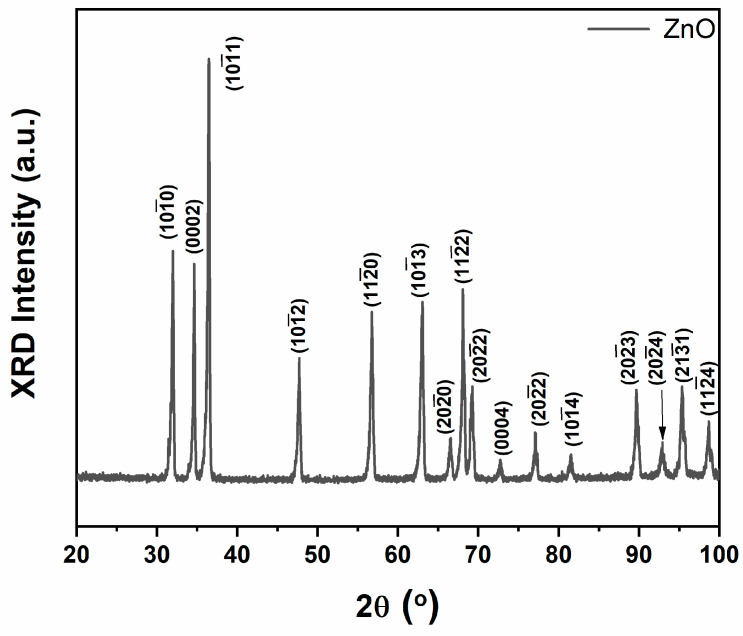
XRD pattern of t-ZnO networks.

In Figure 4, the XRD pattern of the ZnO tetrapodal networks from 20 to 100° is presented. Multiple ZnO diffraction peaks were detected and assigned according to PDF 036-1451. The maximum intensity was observed for peak 10$\bar{1}1$ at 36.46°. The high intensity of the ZnO diffraction peaks compared to the background indicates the good crystallinity of the ZnO tetrapod networks. No Zn peaks were observed, showing the complete oxidation of the Zn particles to ZnO during synthesis [44].

### 3.2. Photo Response at Different Operating Conditions

**Figure 5:**
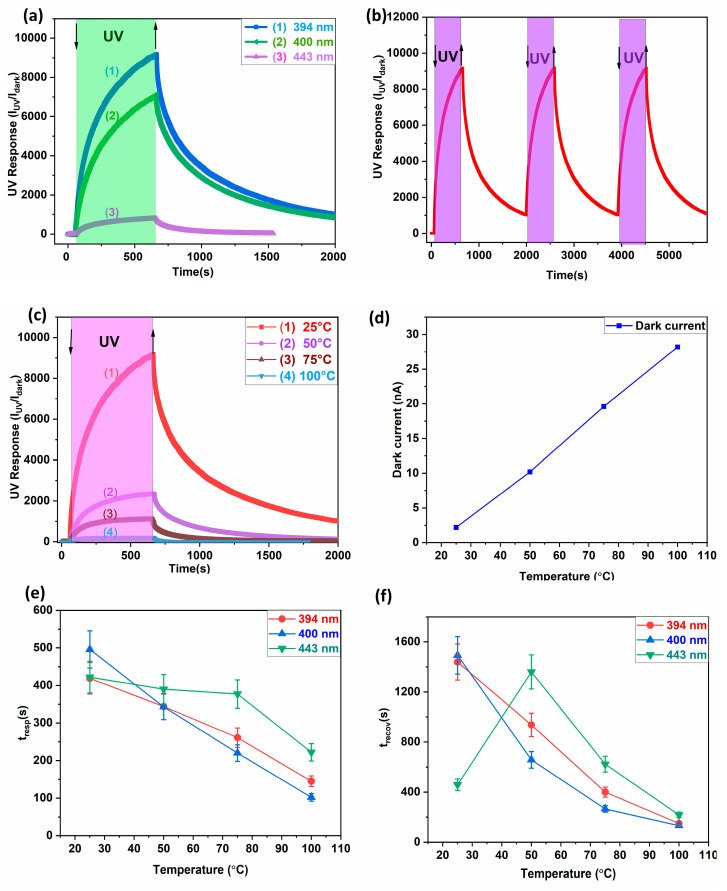
Photo response at 5 V for t-ZnO networks: (**a**) dynamic response at three different photo illumination wavelengths of 394 nm, 400 nm, and 443 nm at 25 °C; (**b**) dynamic response at 394 nm UV illumination at 25 °C for repeatability test; (**c**) dynamic response to 394 nm wavelength at different operating temperatures of 25 °C, 50 °C, 75 °C, and 100 °C; (**d**) effect of operating temperature on dark current or Joule heating effect; and (**e**,**f**) effect of operating temperature on response/recovery time for different photo-irradiation wavelength of 394 nm, 400 nm, and 443 nm.

In this study, UV detection was tested under different operating conditions using two different UV wavelengths: 394 nm and 400 nm, and one wavelength at 443 nm in the visible light spectrum as a reference, as shown in Figure 5a. These results are shown at four different temperatures of 25 °C, 50 °C, 75 °C, and 100 °C, with a bias voltage of 5 V applied. We observed repeatability for 394 nm UV illumination at 25 °C, as shown in Figure 5b.

The UV response decreased with an increasing operating temperature for a temperature range from 25 °C to 100 °C, as shown in Figure 5c. This was due to bandgap narrowing [47]. By increasing the operating temperature, a large number of charge carriers were produced due to the surface desorption of oxygen atoms, leading to an increase in the dark current [48]. Thus, the thermal energy contribution increased the desorption rate and reduced the depletion layer, leading to an increase in dark current. This increase in dark current at higher temperatures reduced the UV response:

(1) $$UV\;response=\frac{I_{UV}}{I_{dark}}$$

where *I_UV_* and *I_dark_* represent the measured current in the presence of photo irradiation and the absence of photo irradiation, respectively.

The response time (*t_resp_*) is defined as the time taken by the sensor to reach 90% of the maximum response value of each cycle. The time taken for the sensor to relax to 10% of the maximum response is called the recovery time (*t_recov_*) [43]. As the operating temperature increased from 25 °C to 100 °C, the photo response time decreased from ~410 s to ~150 s and the recovery time decreased from ~1400 s to ~100 s, as shown in Figure 5e and Figure 5f, respectively. This can be attributed to the faster response–recovery time due to the acceleration of the adsorption–desorption process of surface oxygen atoms at higher temperatures [48,49]. The photodetector based on t-ZnO networks showed a rather slow response and recovery during UV illumination, which can be attributed to the slow adsorption–desorption of oxygen molecules on the sensing surface. Due to the presence of a large number of oxygen vacancies in t-ZnO, the electronic mobility in t-ZnO was enhanced. The PPC effect (persistent photoconductivity) resulted in a high recovery time and slower re-adsorption of oxygen molecules on the surface [49]. The PPC recovery rate increased with an increasing operating temperature, as shown in Figure 5f. The Arrhenius time equation for the ZnO surface in the operating temperature range shows thermally activated behavior:

(2) $$t_{recov}=t_{0}e^{\frac{\Delta E}{kT}}$$

where *t_recov_*, *t*_0_, Δ*E*, *k*, and *T* represent the recovery time at a particular operating temperature, recovery time constant, activation energy barrier, Boltzmann’s constant, and operating temperature, respectively.

Responsivity and external quantum efficiency (EQE) are calculated as shown in Equations (3) and (4), respectively.

(3) $$Responsivity=\frac{I_{UV}-I_{dark}}{opticalpower}$$

(4) $$EQE=\frac{h\cdot c\cdot R}{\lambda \cdot q}$$

where *I_UV_*, and *I_dark_* represent the UV photocurrent current and dark current, respectively, *λ*—illuminated wavelength, *q*—carrier charge, *h*—Planck’s constant, *c*—speed of light, and *R*—responsivity.

**Figure 6:**
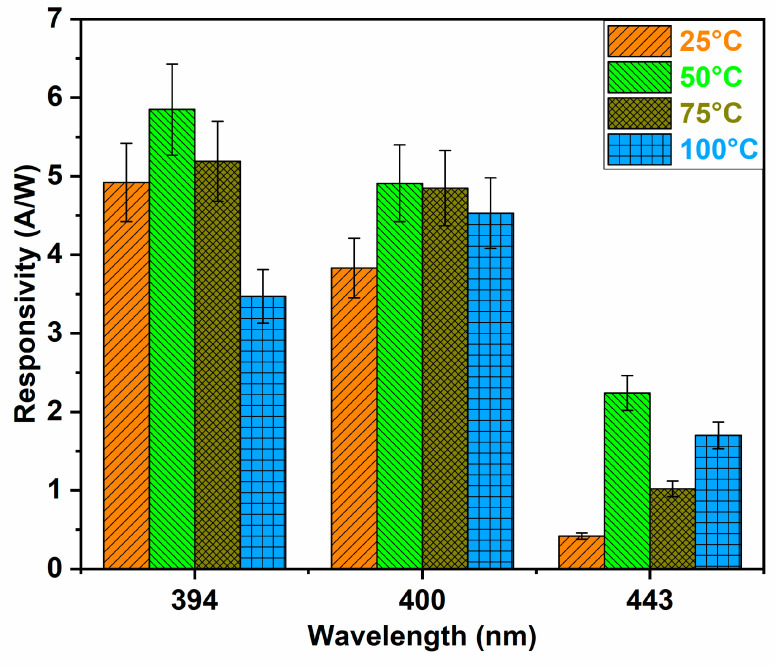
Effect of different wavelengths on responsivity of t-ZnO networks at four different operating temperatures of 25 °C, 50 °C, 75 °C, and 100 °C.

This study showed a high responsivity and EQE for 394 nm illumination at 50 °C, which was maximum against all other operating temperatures, as shown in Figure 6. As the operating temperature increased, the maximum responsivity or EQE shifted towards higher wavelengths [50,51]. For 394 nm UV illumination, the t-ZnO networks exhibited ~5.85 A/W responsivity with an external quantum efficiency of ~18.41 at an operating temperature of 50 °C, which is significant compared to other wavelengths at the same operating conditions. The maximum responsivity at 394 nm UV irradiation can be attributed to the near band-edge emission of ZnO tetrapodal networks [51].

In our case, the maximum responsivity at 50 °C was shown by the sample tested at 394 nm UV illumination, and by increasing the operating temperature from 50 °C to 100 °C, there was a shift in the maximum responsivity from 394 nm to 400 nm, as shown in Figure 6. This redshift in the maximum responsivity can be attributed to the shrinkage of the bandgap energy at higher temperatures [52].

Detectivity (*D*), along with responsivity and quantum efficiency, is one of the key metrics in photodetection. Detectivity is the ability of the photodetector to detect the magnitude of the weakest signal from the light source. Detectivity can be expressed as an equation as follows:

(5) $$D=\frac{R\cdot \sqrt{A}}{\sqrt{2q\cdot I_{dark}}}$$

where *R*, *A*, and *q* represent the responsivity, effective area of the illuminated sample, and charge of an electron, respectively.

Of all the conditions tested, the maximum detectivity was observed with UV illumination at 394 nm, 4.1 μW, and 5 V at 50 °C. With an effectively illuminated sample area of 2.7 cm^2^, the detectivity under the given conditions amounted to be 1.6 × 10^14^$\;\;\frac{\mathrm{cm}\sqrt{\mathrm{Hz}}}{W}$. It was observed that the responsivity and, hence, the detectivity showed higher values at higher applied bias voltages. This can be attributed to the high number of photogenerated charge carriers, i.e., electrons, accelerated by the applied electric field, which contribute to the photocurrent at higher voltages [53].

By increasing the applied bias voltage, both the dark current and the photocurrent increased while the response time decreased [54]. This can be attributed to a stronger electric field, resulting in fast carrier travel. In our case, by increasing the applied voltage, the dark current increased to 1.4 nA, 4.2 nA, and 10.2 nA at 1 V, 3 V, and 5 V, respectively. Similarly, the photocurrent increased to 1.62 μA, 6.89 μA, and 24 μA at 1 V, 3 V, and 5 V, respectively, as shown in Figure 6. This can be attributed to the fact that more photogenerated charge carriers were accelerated by the applied electric field, thereby increasing the photocurrent [53].

Table 1 shows the ZnO-based photodetectors and their corresponding photodetection performances. This comparative study shows the maximum detectivity (1.6 × 10^14^) and EQE (1841.1) for our work. The responsivity was also good and comparatively higher than most of the other reported work. This work attracts more attention due to the weak signal illumination (~0.56 µW).

By increasing the applied bias voltage, the carrier transit time ($\tau_{transit}$) decreased and, hence, the response speed increased, as shown in Equation (6). Khalil et al. confirmed [62] that increasing the applied bias voltage reduces the transit time, and, hence, a faster response occurs for UV-generated charge carriers at a higher bias voltage. Thus, it can be observed that, by increasing the applied voltage (1 V, 3 V, and 5 V), the response time decreased (386 s, 371 s, and 344 s), respectively, which can be attributed to the faster charge carrier moving towards the electrode [63].

(6) $$\tau_{transit}=\frac{l^{2}}{\mu \cdot V}$$

where *μ*, *l*, and *V* represent the mobility of the charge carriers, length of the electrode, and bias voltage respectively.

Table 2 shows the changes in the dark current, UV response, responsivity, photocurrent, and response/recovery rate with an increasing bias voltage at 50 °C for 394 nm.

**Figure 7:**
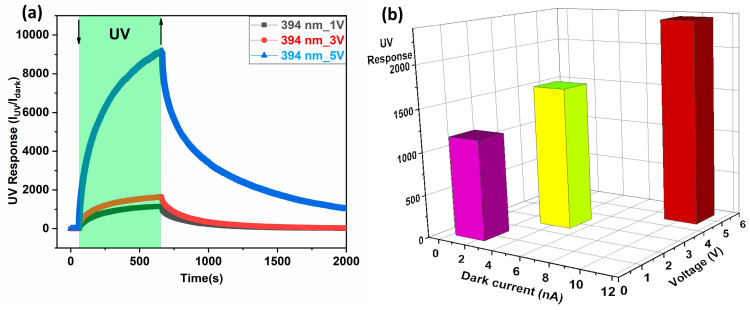
(**a**) Effect of applied bias at three different voltages on UV response to light with 394 nm wavelength at 50 °C; (**b**) 3D figure represents the effect of applied bias voltage on UV response and dark current (red color—5 V, Fluorescent color—3 V, Pink color—1V).

As can be seen in Figure 7, the UV response increased in order (1164, 1644, and 2369) with the applied voltage (1 V, 3 V, and 5 V). This higher UV response with an increasing applied bias voltage can be attributed to an increase in drift velocity. In general, increasing the applied bias voltage expands the space charge region, which can lead to an increase in photocurrent [52]. The non-linear IV characteristics can be observed in Figure 7b and are confirmed by the data in Table 2. This indicated the presence of internal gain amplification, which increased the sensitivity of the device and enabled it to detect weak optical signals. This can be confirmed by calculating the detectivity (~9.7 × 10^13^$\frac{\;\mathrm{cm}\sqrt{\mathrm{Hz}}}{W})$ at 7 V using Equation (5).

The highest UV response of ~9200 was obtained under 394 nm UV illumination at 25 °C with a bias of 5 V applied. This high UV response provided a good signal-to-noise ratio for the illuminated area on the t-ZnO sample. Compared to the photo response of ~800 for visible illumination (443 nm), the UV response was ~12 times higher, resulting in a high UV/visible rejection ratio. This can be attributed to the tetrapod-shaped ZnO network surface, which was more sensitive to UV illumination compared to the visible region [64].

### 3.3. Temperature Sensor

**Figure 8:**
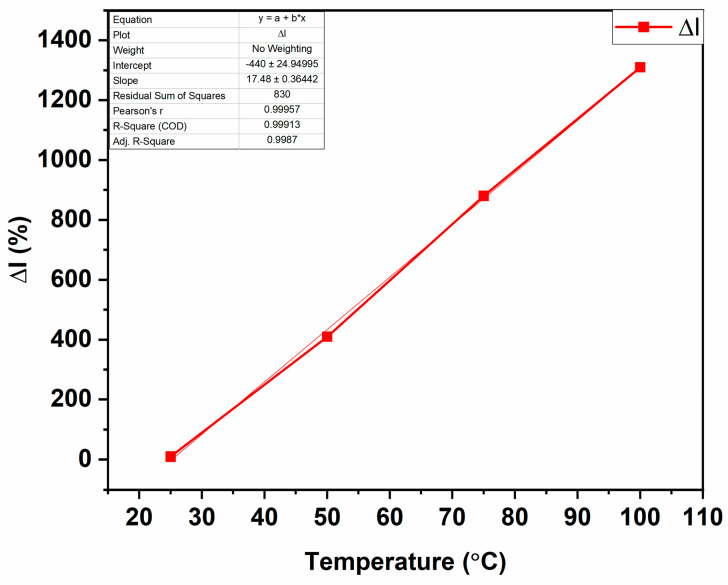
Effect of operating temperature on relative dark current upon exposure of t-ZnO network to light with 394 nm wavelength for the calculation of the temperature sensitivity.

The dark current sensitivity was measured as a function of the operating temperature. The dark current is a good parameter for investigating the temperature sensitivity of a developed structure. The relative dark current of the t-ZnO sample increased linearly with an increasing operating temperature, as shown in Figure 8. In Figure 8, the theoretical and measured relative current (ΔI) are plotted against the operating temperature. The relative current (ΔI) is calculated as shown in Equation (7).

(7) $$\Delta I=\frac{\left(I_{t}-I_{0}\right)}{I_{0}}\times 100\%$$

where $I_{0}$—initial current value and $I_{t}$—final current value at different temperatures. The sensitivity of the sample can be determined by the slope of Figure 8. The sensitivity of the temperature sensor amounted to be 17.48%/°C. The sensitivity was calculated by taking the slope of the linear plot in Figure 8.

As ZnO is an n-type semiconductor oxide, the dark current increased with an increasing temperature [65].

(8) $$\sigma =n\cdot e\cdot \mu_{e}$$

(9) $$n=\sqrt{N_{D}\cdot N_{C}}.{\left(\frac{2\pi \cdot m_{e}\cdot k\cdot T}{h^{2}}\right)}^{\frac{3}{2}}.\exp\left(-\left(\frac{E_{C}-E_{D}}{2k\cdot T}\right)\right)$$

where σ—electrical conductivity at a given temperature, *n*—intrinsic carrier concentration, *μ_e_*—electron mobility, *N_D_*—density of donor states, *N_C_*—density of states in the conduction band, *m_e_*—effective mass of an electron, and *E_C_* and *E_D_* represent the bottom edge energy of the conduction band and the donor level energy, respectively.

Equations (8) and (9) show that the increase in the carrier concentration occurred, to some extent, by increasing the operating temperature, and, hence, the electrical conductivity. It was observed that the dark current increased with both an increasing temperature and increasing bias voltage. This may have been due to thermal generation and the high carrier mobility of the charge carriers, respectively [66]. Figure A3 shows the Arrhenius plot of the t-ZnO networks. The slope of the Arrhenius plot can give the activation energy (*E_a_*) from the Boltzmann constant (*k_B_*):

(10) $$Slope=\frac{E_{a}}{k_{B}}$$

By linear fitting, the slope amounted to be (~4744.26) with a 10% error. From Equation (10), the activation energy amounted to be 0.4 eV with a 10% error. This activation energy may be related to the oxygen interstitial (O_i_) above the valence band [67,68]. It may lead to the possibility of transitioning from the conduction band to O_i_ corresponding to a wavelength of 419 nm.

### 3.4. Gas Sensing

Gas sensors based on t-ZnO networks for the detection of different environmental biomarkers under different operating conditions are presented in this study.

The gas response (*S*) was determined by using the ratio of currents in air (*I_air_*) and during gas exposure (*I_gas_*) [69]:

(11) $$S=\frac{I_{gas}}{I_{air}}$$

**Figure 9:**
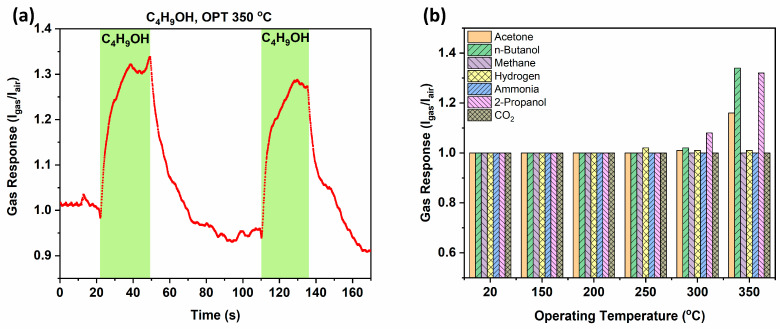
(**a**) Dynamic response of t-ZnO networks-based sensor for 100 ppm of n-butanol at an operating temperature of 350 °C and (**b**) response value of the developed device at different temperatures from 20 °C to 350 °C for a series of gases and VOCs with concentration of 100 ppm.

At low temperatures ranging from 20 °C to 250 °C, the investigated t-ZnO networks-based gas sensor showed no response and selectivity to any of the test gases. Responses to VOCs as biomarkers/analytes were observed at temperatures above 250 °C, as shown in Figure 9b. The maximum response of ~1.3–1.35 was observed for 100 ppm n-propanol and 100 ppm n-butanol. A lower response was also observed for 100 ppm of acetone. No response was observed for other analytes such as methane, hydrogen, ammonia, and CO_2_. At an operating temperature of 350 °C, a maximum response of ~1.35 with a high response/recovery rate was observed repeatably, as presented in Figure 9a. The dynamic response for n-propanol is presented in Figure A2. The response/recovery times for 100 ppm n-butanol were determined with dynamic response cycles of ~12 s/16 s, respectively.

In Figure 9a, a small baseline drift can be seen after each gas desorption cycle, which can be attributed to environmental changes or more instability factors [70]. However, the sensing response remained intact after each cycle, demonstrating the good repeatability exhibited by the t-ZnO networks for *n*-butanol sensing.

### 3.5. UV Sensing Mechanism

**Figure 10:**
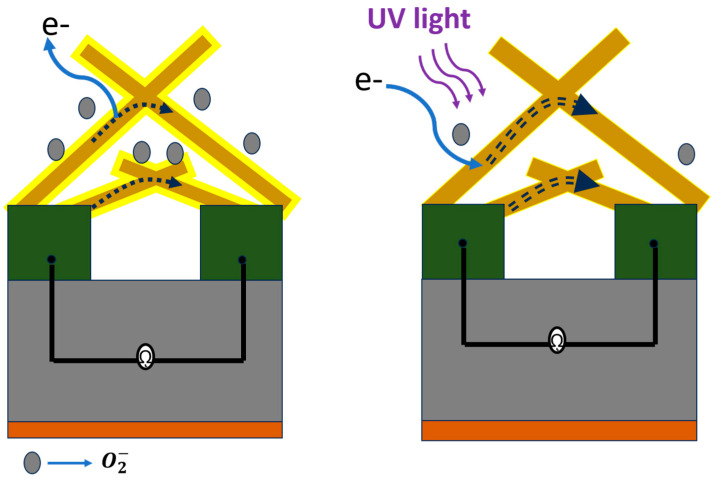
Schematic representation of UV-sensing mechanism of t-ZnO networks.

A schematic representation of the sensor is shown in Figure 10. It explains the surface dynamics proposed of the ZnO sensing surface based on the occurrence of the adsorption–desorption of oxygen molecules on the ZnO surface. The UV-sensing mechanism of ZnO is based on changes in physical properties by the reversible adsorption–desorption of oxygen molecules on ZnO surfaces [71]. Due to the adsorption of oxygen molecules on the surface, it reacts with free electrons from the conduction band, as shown in Equation (12).

(12) $$O_{2\left(\mathrm{gas}\right)\;}+{\;e}^{-}\;\to {O_{2}}_{\left(\mathrm{ads}.\right)}^{-}$$

The adsorbed oxygen species on the ZnO surface formed the potential barrier around the grain boundaries. This led to an increase in the space charge region and a reduction in conductivity on the surface of the t-ZnO. Depending on the temperature, the pressure, and the chemical environment, ZnO possesses several native neutral oxygen vacancies. Illuminating the sensing surface with UV light (*E*_UV_ > *E*_g_) led to the generation of electron–hole pairs, as shown in Equation (13). The photogenerated holes became trapped at the originally neutral oxygen vacancies that lay within the band gap region [72]. As a consequence, free excess electrons with a superior lifetime were generated in the conduction band, which significantly increased the electrical conductivity of ZnO. By increasing the temperature, the depletion layer width was reduced due to the enhancement of the desorption rate, which led to more carrier generation. This may lead to an increase in dark current and affect the UV response.

(13) $$\mathrm{hv}\to e^{-}+{\;h}^{+}$$

(14) $$O_{2\left(\mathrm{ads}.\right)}^{-}+{\;h}^{+}\;\to O_{2\left(\mathrm{desorp}.\right)}$$

where $O_{2\left(\mathrm{gas}\right)\;},{O_{2}}_{\left(\mathrm{ads}.\right)}^{-}$, and $O_{2\left(\mathrm{desorp}.\right)}$ represent an oxygen gas molecule, adsorbed oxygen ion, and desorbed oxygen molecule, respectively.

These adsorption–desorption dynamics affect the response–recovery rate of charge carriers. Due to the PPC (persistent photoconductivity) effect, slow carrier dynamics during the adsorption–desorption process led to a slow rise/decay rate.

### 3.6. Gas-Sensing Mechanism

**Figure A1:**
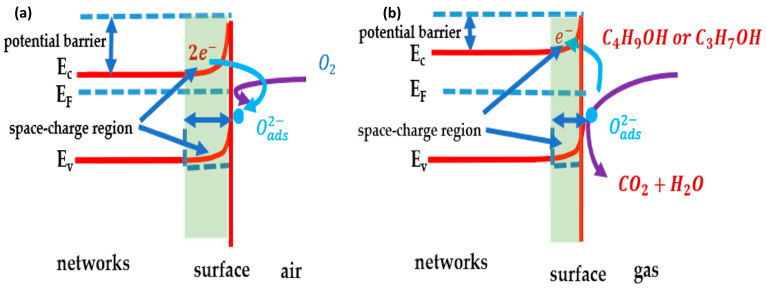
Schematic of gas-sensing mechanism of ZnO tetrapodal networks-based sensing sample (**a**) in air and (**b**) in n-butanol or n-propanol.

**Figure A2:**
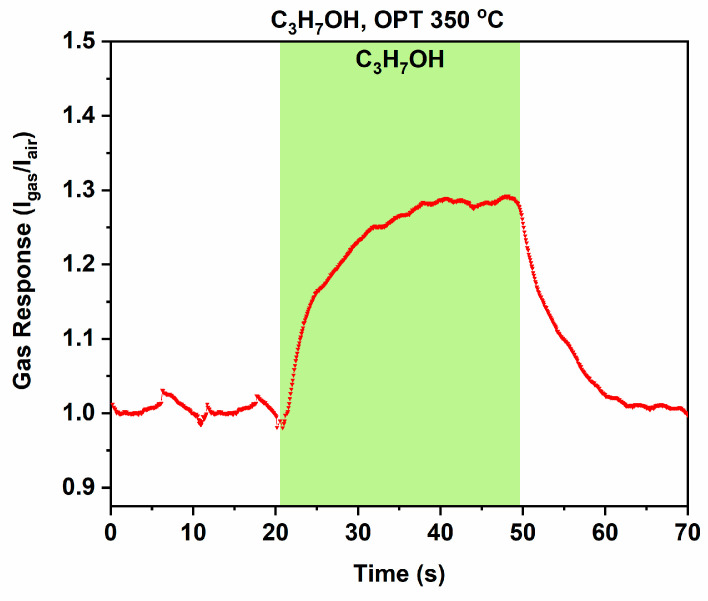
Dynamic response of t-ZnO networks-based sensor for 100 ppm of n-propanol at an operating temperature of 350 °C.

**Figure A3:**
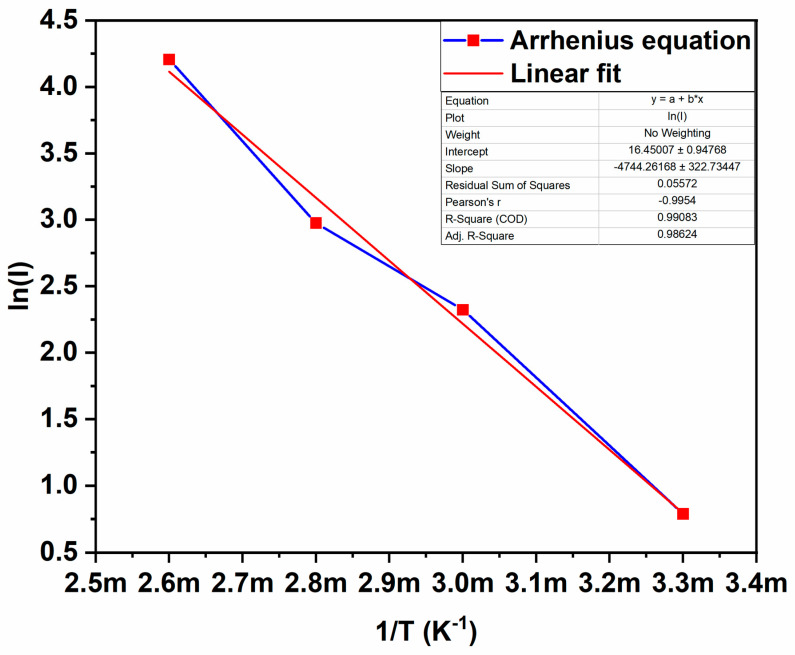
Arrhenius plot of t-ZnO networks with an activation energy of *E_a_* = 0.4 eV.

The detection of n-butanol or n-propanol vapor/molecules can be explained on the basis of the conductance principle and a model by Wolkenstein [73,74], as shown in Figure A1. Before the injection of the target analyte, oxygen species depending on the surrounding temperature are adsorbed on the surface (O_2(ads.)_) of the t-ZnO networks. The oxygen molecules on the surface can effectively react with electrons and form oxygen ions (2O^−^), leading to the formation of a thick electron depletion region or space charge region, as shown in Figure A1a. This effect causes the free electrons on the surface to move towards the bulk, resulting in a decrease in conductivity on the sensor surface. Because this conductivity is related to the cross-section of the conductor, and the conducting cross section becomes smaller when the oxygen ions adsorb, the conductivity decreases. After the injection the target analyte (i.e., butanol or propanol), this analyte reacts with the adsorbed oxygen species and releases the electrons back to the surface, leading to an increase in the conductivity cross-section, and hence, the conductivity increases. All this can also be expressed in equation form, as shown in Equations (15)–(17) [74,75,76]:

(15) $$O_{2\;\left(\mathrm{gas}\right)}↔O_{2\left(\mathrm{ads}.\right)}$$

(16) $$O_{2\left(\mathrm{ads}.\right)}+2e^{-}\;\to 2O_{\left(\mathrm{ads}.\right)}^{-}$$

(17) $${\left(C_{4}H_{9}\mathrm{OH}\right)}_{\mathrm{ads}.}+12O_{\mathrm{ads}.}^{-}\to 4{\mathrm{CO}}_{2}+5H_{2}O+12e^{-}$$

## 4. Conclusions

We demonstrated a multifunctional three-in-one sensor based on t-ZnO networks. The sensor is capable of VOC and temperature sensing and UV-A photodetection for biomedical applications. In SEM measurements, the tetrapodal morphology of the ZnO networks grown by FTS was observed. XRD studies showed the complete oxidation of Zn. The high crystallinity of the t-ZnO networks was confirmed by the presence of typical XRD reflexes. The t-ZnO networks exhibited the highest UV detection at 394 nm illumination, with an external quantum efficiency of ~19 and a responsivity of ~5.85 A/W at 50 °C. Additionally, there was an excellent photo response at 25 °C with a response value of ~9200, which was ~12 times higher than that at visible illumination (443 nm). The voltage variation indicated that a higher applied bias voltage resulted in better response, with a higher responsivity and UV current observed at 5 V compared to the other tested bias voltages, such as 3 V and 1 V. The t-ZnO networks showed an excellent repeatability, selectivity, and sensitivity for UV sensing, especially at a 394 nm UV wavelength. A monotonic increase in the response–recovery rate was observed by increasing the operating temperature. Low-temperature UV detection (394 nm) was ~43 times higher at 25 °C than at 100 °C. This can be attributed to an increase in the dark current at higher temperatures. The maximum detectivity was 9.7 × 10^13^$\;\frac{\;\mathrm{cm}\sqrt{\mathrm{Hz}}}{W}$ for UV illumination (394 nm, 50 °C, 7 V). t-ZnO networks can detect 100 ppm n-butanol and n-propanol at temperatures above 250 °C. The sensor exhibited a low recovery time of ~16 s for n-butanol, demonstrating its potential as a VOC detector for medical and bioengineering applications. On the other hand, the t-ZnO networks demonstrated temperature sensitivity in the temperature range tested from 25 °C to 100 °C, with a sensitivity of 17.48%/°C. Multifunctional sensors that detect VOCs and UV-A and provide information on operating temperature are very useful for monitoring cell culture media properties in biomedical applications. This is particularly important when studying the presence of different alcohol groups at different temperatures. The prepared t-ZnO networks-based sensors enable effective fast local temperature monitoring for lab-on-a-chip applications. Furthermore, they enable the monitoring of UV exposure, which helps to prevent wrinkles and skin cancer. The fundamental information about the activation energy was extracted from the Arrhenius plot (~0.4 eV), which corresponds to oxygen interstitials (O_i_) above the valence band. Further research is required to increase the selectivity for different alcohol groups at lower detection limits using catalytic effects in the presence of appropriate catalysts.

## Figures and Tables

**Table 1:** ZnO-based photodetectors and their corresponding device performances.

Sensing Material	λ (nm)	Intensity (mW/cm^2^)	R (A/W)	D (Jones)	EQE (%)	Applied Bias (V)	Reference
ZnO NRs/PANI	400	-	0.039	-	12.3	5	[55]
t-ZnO with complex arms	365	50	2.1	-	713.4	3	[56]
ZnO/PANI NC	365	12,740	1.43	-	485.8	0	[57]
Cu_2_O/ZnO/PVK	360	24.9 µ	13.28	1.03 × 10^13^	-	−0.1	[58]
ZnO/Au/PEDOT:PSS	380	-	0.181	1.39 × 10^11^	59	8	[59]
ZnO/(Cu:ZnO)	395	4	4.5 m	-	1.4	0.5	[60]
ZnO:Mn NRs	400	-	1.25 m	-	0.3	1	[61]
t-ZnO networks	394	0.56 µ	5.85	1.6 × 10^14^	1841.1	5	This work

**Table 2:** Effect of bias on dark current, UV response, responsivity, *I*_UV_, and response/recovery time to 394 nm wavelength at 50 °C.

**Bias Voltage (V)**	**Dark Current (nA)**	**Response**	**Responsivity (A/W)**	***I*_UV_ (μA)**	**Response/Recovery Time (s)**
1	1.4	1164	0.394	1.62	386/514
3	4.2	1644	1.680	6.89	371/614
5	10.2	2369	5.85	24	344/937

## Data Availability

Data are contained within the article.
